# Molecular Profiling Reveals Common and Specific Development Processes in Different Types of Gynecologic Cancers

**DOI:** 10.3389/fonc.2020.584793

**Published:** 2020-10-29

**Authors:** Yuanli Guo, Junfeng Liu, Jiaqi Luo, Xiaobin You, Hui Weng, Minyi Wang, Ting Ouyang, Xiao Li, Xiaoming Liao, Maocai Wang, Zhaoji Lan, Yujian Shi, Shan Chen

**Affiliations:** ^1^ Department of Gynaecology and Obstetrics, The Sixth Affiliated Hospital, Sun Yat-sen University, Guangzhou, China; ^2^ Department of Pathology, The First Affiliated Hospital, Sun Yat-sen University, Guangzhou, China; ^3^ Department of Research, Top Gene Tech (Guangzhou) Co., Ltd., Guangzhou, China

**Keywords:** cervical cancers, endometrial cancers, ovarian cancers, integrated molecular analysis, TCGA, PI3K-Akt-mTOR signaling, mismatch repair, cilium organization

## Abstract

**Background:**

Gynecologic cancers have become a major threat to women’s health. The molecular biology of gynecologic cancers is not as well understood as that of breast cancer, and precision targeting is still new. Although viewed collectively as a group of cancers within the female reproductive system, they are more often studied separately. A comprehensive within-group comparison on molecular profiles is lacking.

**Methods:**

We conducted a whole-exome sequencing study of cervical/endometrial/ovarian cancer samples from 209 Chinese patients. We combined our data with genomic and transcriptomic data from relevant TCGA cohorts to identify and verify common/exclusive molecular changes in cervical/endometrial/ovarian cancer.

**Results:**

We identified shared molecular features including a COSMIC signature of deficient mismatch repair (dMMR), four recurrent copy-number variation (CNV) events, and extensive alterations in PI3K-Akt-mTOR signaling and cilium component genes; we also identified transcription factors and pathways that are exclusively altered in cervical/endometrial/ovarian cancer. The functions of the commonly/exclusively altered genomic circuits suggest (1) a common reprogramming process during early tumor initiation, which involves PI3K activation, defects in mismatch repair and cilium organization, as well as disruption in interferon signaling and immune recognition; (2) a cell-type specific program at late-stage tumor development that eventually lead to tumor proliferation and migration.

**Conclusion:**

This study describes, from a molecular point of view, how similar and how different gynecologic cancers are, and it provides a hypothesis about the causes of the observed similarities and differences.

## Introduction

Gynecologic cancers have been estimated to claim more than 1.3 million (16.5% of all cancers in women) new cases worldwide in 2018, according to world cancer data (1). Surgical resection + chemotherapy and/or radiotherapy (in some advanced cases, only chemo/radiotherapy) remains as the mainstream of gynecologic cancer treatment. The molecular biology of this group of cancers is yet to be fully established, posing difficulties in molecular subtyping and precision targeting. Although the discovery of the predisposing effects of HPV infection has greatly improved the diagnosis and prevention of cervical cancer (CC), there is still a lack of effective screening methods for other gynecologic cancers, mainly endometrial cancer (EC) and ovarian cancer (OC).

How similar are different types of gynecologic cancers? And how are they distinguished from each other? On one hand, they all originate from the Mullerian ducts and all reside within the female reproductive system, which is under the regulation of female hormones (2). On the other hand, they arise from different cell types, having different clinical outcomes (survival, risks of recurrence/metastasis) and are thought to be caused by different mechanisms. For example, squamous cell carcinoma accounts for most of CC, while adenocarcinoma (from glandular cells) is the major histotype of EC, and serous cell carcinoma is mostly seen in OV. Unlike CC, which is most likely to be caused by HPV infection, the majority of EC is thought to be associated with long-term irritations by imbalanced female hormones. OV is generally believed to be the most aggressive gynecologic cancer type, and the cause of OV is controversial, with recent hypotheses suggest a non-ovarian origin (fallopian tube epithelium) (3). However, we have not seen many studies addressing the above questions from a molecular point of view. Although the TCGA molecular study on “Pan-Gyn” (gynecologic + breast) cancers (2) is the one with the largest sample size and the most comprehensive platforms, it included a large number of breast cancers (accounting for more than 40% of total samples) into the Pan-Gyn category, which may have affected the characterization of gynecologic cancer samples. Another study with a relatively small sample size (n = 117, 68 OC + 32 CC + 17 EC) focused on calculating tumor mutational burden (TMB) in Chinese gynecologic cancer patients (4). The study showed that EC have a higher median TMB than CC or OC, and mutations in PTEN, TSC2, or POLE are associated with increased TMB. To the best of our knowledge, a clear summary or conclusion of what molecular features are shared/exclusive in various types of gynecologic cancers is absent in existing literature.

While it is important to find out what the shared/exclusive molecular features are, it is even more important to understand why they are so. What intrinsic mechanisms drive these closely related cell types to develop into cancers with distinct phenotypes? Are there any common processes involved during the development of different types of gynecologic cancers, as reflected by their close distances? We believe the answers to these questions will help advance our understandings of the development of gynecologic cancers.

We conducted a whole-exome sequencing study in a total of 209 (74 CC, 68 EC, and 67 OC) Chinese gynecologic cancer patients. We examined the mutation landscape of the samples and validated our results with genomic and transcriptomic data from TCGA gynecologic cancer cohorts, namely TCGA-CESC (5), TCGA-UCEC (6), and TCGA-OV (7). Significant consistency was observed between the Chinese and the TCGA data. Similar mutation patterns were found among CC, EC, and OC at all levels (chromosomal changes, mutation signature, signaling pathways, and biological processes), indicating a common reprogramming process of cells at early stages of tumor development. We also identified transcription factors (TFs) and their relevant pathways that were exclusively altered in each cancer type, which suggest a possible cell-type specific program that further makes each cancer type form into shape.

## Materials and Methods

### Samples

We initially included surgically resected tumor samples from 263 sporadic gynecological cancer patients treated at The Six Affiliated Hospital of Sun Yat-sen University and The First Affiliated Hospital of Sun Yat-sen University from January 2017 to June 2019. The inclusion criteria for patients were (1) aged 20–82 years old; (2) initial diagnosis of primary cancer, confirmed by post-operative pathology; (3) previously untreated; (4) over 50% tumor cell content observed in hematoxylin and eosin stain slides under microscope. The exclusion criteria: (1) metastatic cancer; (2) ambiguous pathology; (3) accompanied by malignant tumors of other organs; (4) failed sample quality or insufficient amount of sample for experiment. Another two samples of rare cancer types (vaginal cancer and sarcoma) were excluded due to too small sample sizes. The final data set (n=209) included 74 cervical cancer (CC) cases, 67 ovarian cancer (OC) cases, and 68 endometrial cancer (EC) cases. Clinical information of each case was extracted from medical records, including age of diagnosis, classification and staging (TNM), progression status, and HPV status detected using HPVDetector (8). Informed written consent was obtained from each patient. This study was approved by the Ethics Committee of The Sixth Affiliated Hospital of Sun Yat-sen University. All procedures performed within this study were done in accordance with the Chinese ethical standards and with the 2008 Helsinki declaration.

### Whole-Exome Sequencing

All tumor tissue samples were sent to TopGene Medical Laboratory (Zhongshan, China) for whole-exome sequencing. Genomic DNA extraction was performed using Mag-bind blood and tissue DNA HDQ 96 kit (Omega Bioservices, Norcross, GA, USA), according to the manufacturer's instructions. A UV spectrophotometer (NanoDrop Technologies, Wilmington, DE, USA) was used to check DNA quality. DNA quantification was performed with Qubit fluorometer 3.0 (Thermo Fisher Scientific, Waltham, MA, USA). Exome capture from the genomic DNA was performed with the AIExomeV1 panel (iGeneTech, China). PCR products were subjected to quality check with LabChip GX Touch24 (PerkinElmer). Pair-end sequencing was performed using MGISEQ-2000RS. The average depth of each sample was ~100X and the read length was 150bp.

### Data Processing and Mutation Analysis

Raw sequencing reads QC and filtering were done with Fastp (9). Read mapping to human genome hg19 was performed using BWA MEM (version 0.7.15-r1140) (10). GATK4 (11) were used for reads processing and the generation of base-quality recalibrated bam files. Somatic variants were first detected using GATK4 Mutect2; these variants were then further verified with samtools mpileup (12) and SomVarIUS (13) (the variant must also be detected by at least one of the two callers); variants with population allele frequency > 0.05 were excluded from the list. Somatic variants with allele fraction < 5% were filtered to reduce false discoveries caused by lack of matched normal and sequencing errors. Germline variants were called using GATK4 Haplotype Caller, and putative germline variants were separately marked. Driver gene analysis was performed using MutSigCV (14). Copy number variation (CNV) was called using GATK4 with a panel of normal, made of 32 normal tissue samples. Default threshold (2.0 z-score of non-log2 copy ratio) was used for the calling. The raw segment files generated by GATK4 CNV caller were then used as input for GISTIC2.0 (15) to calculate significant copy-number alterations with a threshold of ±0.3.The R package maftools (16) was used for the visualization of mutated genes and calculation of differentially mutated genes with Fisher’s Exact Test (p<0.05). Genomic data of TCGA were downloaded from https://portal.gdc.cancer.gov/, and transcriptomic and survival data were downloaded via the RTCGA R package. Enrichment analysis was done using the online tool WebGestalt (17).

## Results

Demographic information of the 209 patients is summarized in **Supplementary Table S1**, which includes histology subtypes, age of diagnosis (ranged from 22–82, mean±SD:51.8±10.3, median: 52), TNM staging, HPV status, and tumor cell differentiation status.

### Mutation Landscape of Gynecologic Cancers

**Figure 1:**
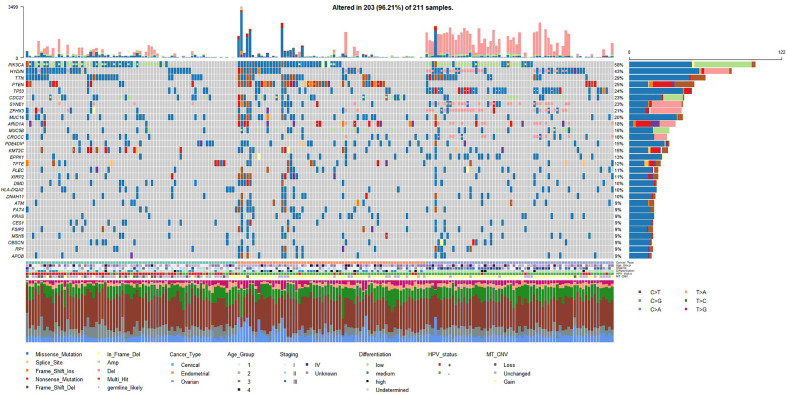
Mutation landscape represented by top 30 frequently mutated genes (small-scale mutations + copy number variations) in the studied gynecologic cancer cohort (n=209). Both small-scale mutations and CNV (Amp/Del) were taken into account. Annotations include cancer type of each sample, age groups (age < 45 - group 1; 45 ≤ age < 55 - group2; 55 ≤ age < 65 - group 3; age ≥ 65 - group 4), TNM overall staging, tumor cell differentiation status, HPV status, and mitochondrial copy number variation (MT_CNV). Likely germline mutations are highlighted with white dots.

The mutation landscape of the studied Chinese gynecologic cancer cohort as represented by top frequently mutated genes are shown in **Figure 1**. We found that EC have the highest mean number of small-scale mutations but the lowest average frequency of CNV; CC have the lowest small-scale mutations and medium frequency of CNV, while OC samples harbor most CNV events. The cancer driver PIK3CA was frequently mutated in all three cancer types (122/209, 58%). Among the frequently altered were other known tumor-related genes such as PTEN (25%), TP53 (24%), CDC27 (23%), ZFHX3 (22%), MUC16 (20%), ARID1A (18%), KMT2C (15%), KRAS (9%), and BRCA2 (9%). We found TP53 mutated in 49% of the OC samples, PTEN mutated in 51% of EC samples, which are consistent with findings in the previous Chinese study (4). The ovarian cancer biomarker MUC16 (also known as CA125) was mutated even more frequently in EC (20%) and CC (29%) samples (compared to 12% in OC), suggesting a common role of MUC16 among gynecologic cancers. Interestingly, a novel gene HYDIN (43%, 90/209) was found highly mutated in all three cancers. The gene has previously been associated with defects in cilia motility but rarely reported in cancer studies. We have further confirmed the HYDIN small-scale mutations with Sanger sequencing.

**Figure 2:**
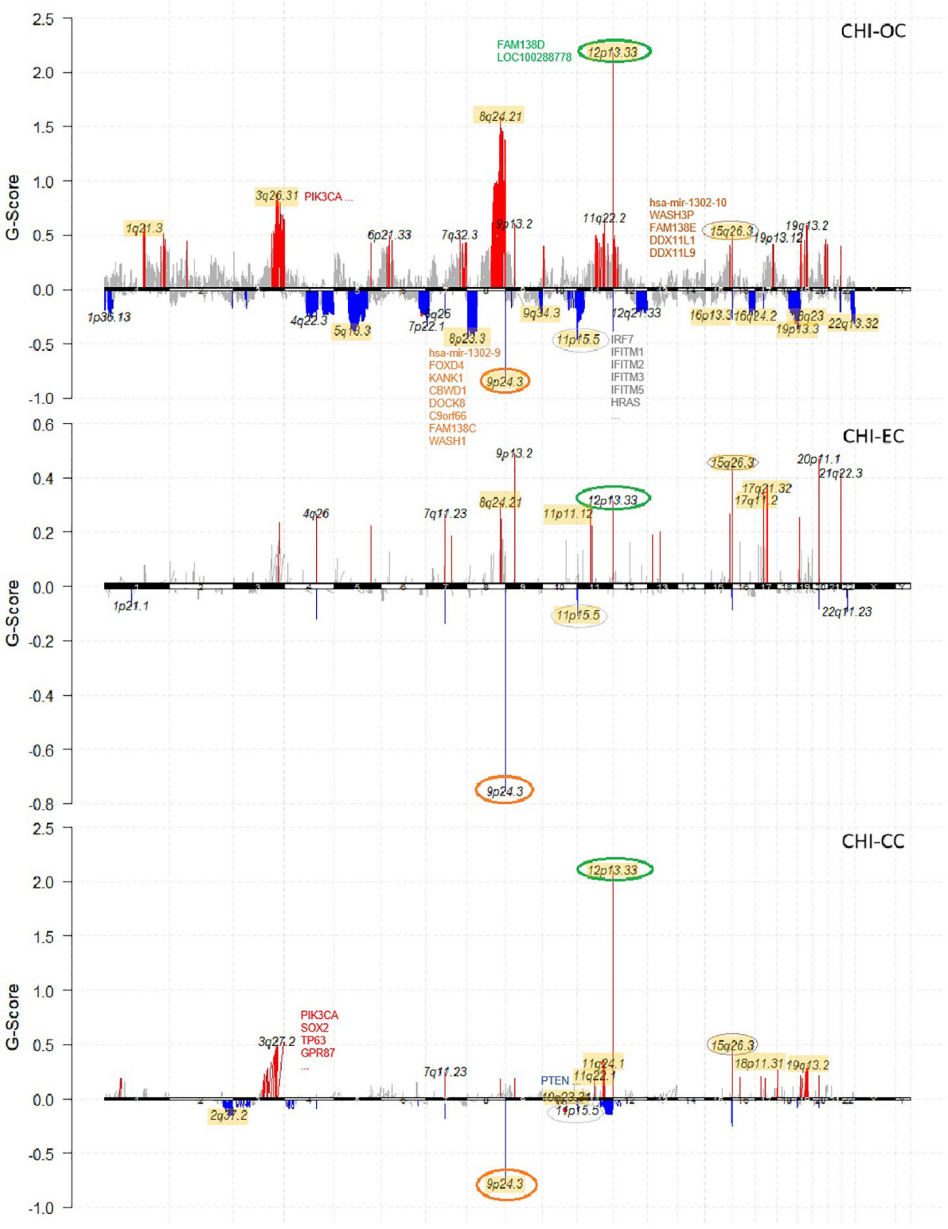
Chromosome plots showing recurrent CNV events identified by Gistic2 in each cancer type (OC, EC, CC) of the studied cohort. Amplifications are colored in red and deletions colored in blue. Common CNVs that occurred in all three cancer types are highlighted with circles. For each cancer type, the results are compared with the corresponding TCGA cohort, and all the overlapping recurrent regions are highlighted in yellow background.

Recurrent CNV events for each cancer type were identified with GISTIC2.0 (q value < 0.05). OC differed from the other two cancer types by having more peaks and much wider peaks, indicating large-scale instability of the genome (**Figure 2**). Four focal CNV events seemed to occur recurrently in all three cancer types at 12p13.33 (amplification; 75/209, 36%), 15q26.3 (amplification; 33/209, 16%), 9p24.3 (deletion; 69/209, 33%), and 11p15.5 (deletion; 47/209, 22%). The recurrently amplified regions include genes encoding retinal proteins FAM138D (12p13.33) and FAM138E (15q26.3), while the recurrently deleted regions include tumor-suppressor genes, e.g., DOCK8 and KANK1, as well as genes that participate in interferon alpha/beta (IFN-α/β) signaling (IRF7, IFITM1/2/3/5). In addition, PIK3CA (3q26.32) and CDC27 (also known as MLL3; 17q21.32) amplifications, HYDIN and ZFHX3 (16q24.2) deletions, SYNE1 (6q25.3) deletions, and ARID1A and CROCC (1p36.13) deletions frequently occurred in OC samples; PIK3CA and CDC27 were also frequently amplified in CC and EC (**Figure 1**).

Driver gene analysis was performed on each cancer type (**Supplementary Table S2**). We confirmed TP53 (q< 0.01) as a significantly mutated gene (SMG) for all 3 cancer types; PIK3CA (q<10-11) as SMG for CC and EC; KRAS (q < 0.1) as SMG for EC and OC; PTEN, PIK3R1, and ARID1A (q<10-3) as SMGs for EC. We also identified PDE4DIP, ZNF285, CDC27, CES1 (q< 0.05) as novel SMGs for CC; CDC27, CYP2D6, UGT2B11 (q< 0.1) as novel SMGs for EC; VHL and ZNF285 as novel SMGs for OC.

### Distinguishing Characteristics Among Gynecologic Cancers, Validated by TCGA Data

**Table 1** summarizes some features that characterize similarities and differences among the three gynecologic cancer types of the studied cohort, validated with genomic and/or transcriptomic data from the gynecologic cohorts of TCGA. Considerable horizontal similarities were found between TCGA vs Chinese for each cancer type, especially for EC with 60% overlap on top10 frequently mutated genes. The Chinese and TCGA data together revealed that EC have the highest average mutation load while OC have the highest average CNV frequency. All three cancer types contained a group of samples with COSMIC (v3) mutation signature indicative of deficiency in mismatch repair (dMMR); a group of CC samples were enriched with signature of APOBEC mutagenesis, while EC differed by having a group with mutation signature indicating defects in the polymerase POLE. The composition of COSMIC signatures for each cancer type were highly similar between Chinese and TCGA data, except TCGA-OV having a group with signature of homologous recombination repair deficiency and CHI-OC with a group of unknown signature. Note that TCGA-OV only consisted of high grade serous carcinoma, whereas the histotype composition of CHI-OC was much more complicated (**Supplementary Table S1**). The distinct histotype composition of the two OV cohorts may explain the dramatic difference in TP53 mutation prevalence (~90% in TCGA-OV and ~50% in CHI-OC) as well as in other molecular features (**Table 1**). Hence the molecular profiles of TCGA-OV and CHI-OC may be incomparable.

**Table 1:** Summary of genomic characteristics of CC, EC, and OC.

	CERVICAL CANCER		ENDOMETRIAL CANCER		OVARIAN CANCER	
	CHI-CC	TCGA-CESC	CHI-EC	TCGA-UCEC	CHI-OC	TCGA-OV*
Average Mutation Load (per tumor)	low (90)	medium (158)	high (262)	high (862)	medium (113)	low (109)
Average CNV Frequency (per tumor)	medium (174)	low (88)	low (71)	medium*	high (834)	high*
Top 10 Mutated Genes	PIK3CA(66%), HYDIN(39%), TTN(34%), CDC27(22%), MUC16(20%), KMT2C(18%), PDE4DIP (15%), HLA-DQA2(14%), PTEN(14%), CES1(12%)	PIK3CA(42%), TTN(31%), KMT2C(19%), MUC16(17%), MUC4(16%), KMT2D(15%), PTEN(13%), DMD(13%), FLG(13%), EP300(13%)	PIK3CA(53%), PTEN(51%), HYDIN(32%), MUC16(29%), CDC27(28%), TTN(28%), PIK3R1(24%), ARID1A(22%), PDE4DIP(22%), SYNE1(21%)	PTEN(57%), PIK3CA(48%), TTN(44%), ARID1A(43%), TP53(36%), MUC16(30%), PIK3R1(30%), KMT2D(27%), CTCF(24%), CSMD3(24%)	HYDIN(58%), PIK3CA(55%), TP53(49%), MUC6(46%), SYNE1(43%), VPS13D(28%), CROCC(27%), EPPK1(24%), TTN(24%), MUC5B(18%)	TP53(88%), TTN(34%), CDKN2A(32%), CCNE1(20%), PIK3CA(18%), CCND2(15%), NF1(12%), KRAS(11%), NOTCH3(11%), RB1(10%)
COSMIC v3 Signature Clusters	APOBEC cytidine deaminase (SBS2); Defective DNA mismatch repair (SBS6)	APOBEC cytidine deaminase (SBS2, SBS13); Defective DNA mismatch repair (SBS1)	Defects in polymerase POLE (SBS10a); Defective DNA mismatch repair (SBS6, SBS26)	Defects in polymerase POLE (SBS10a); Defective DNA mismatch repair (SBS6, SBS44)	Unknown signature (SBS5); Defective DNA mismatch repair (SBS6)	Defects in HR (SBS3); Defective DNA mismatch repair (SBS6)
Exclusively Altered Pathway	**SOX2-TP63** mediated cell survival and pluripotency; **SHOX2**-TGFBR1 mediated EMT; **EAF2**-WNT3A mediated anti-apoptosis		**PBXIP1/HPIP** mediated anti-apoptosis, proliferation and EMT; **CREB3L4**-DNAJC12 mediated proliferation and invasion		**HSF1**-mediated cell survival (RBM23, HSPA8) and EMT (MTA1); **FOXH1**-NODAL signalling for pluripotency maintenance via NANOG; GATA6-**ZFPM2** mediated androgen biosynthesis	
Commonly Altered Components	Cilium organization; PI3K-Akt-mTOR signaling					

*Average CNV count numbers for TCGA-UCEC and TCGA-OV are not mentioned in publications. For each cancer type, genes that are overlapping between Chinese and TCGA are underlined. Transcription factors highlighted with bold text in the “Exclusively Altered Pathway” row are those first identified in the Chinese genomic data and then validated with TCGA transcriptomic data. The rest of the genes in this row are known interacting/downstream molecules that showed co-altered expression patterns to these TFs in TCGA transcriptomic data. Note that TCGA-OV only contains high-grade serous carcinoma samples, while CHI-OC have a more complicated composition, therefore the molecular profiles of the two ovarian cancer cohorts may be incomparable.

**Figure 3:**
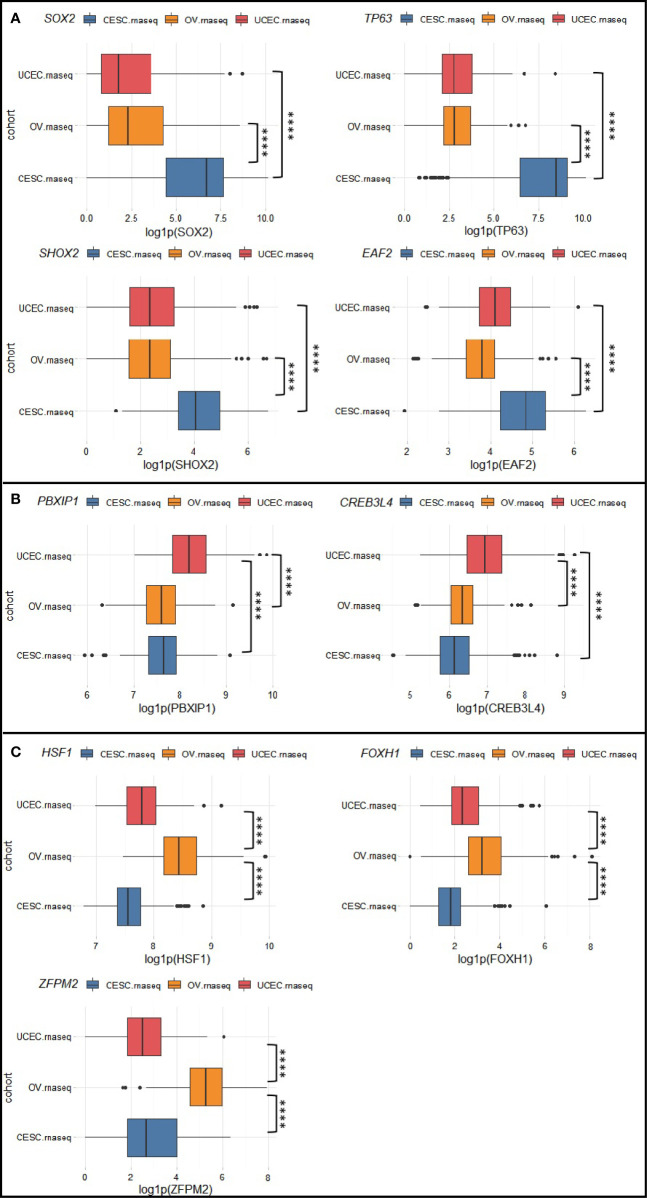
Exclusively altered transcription factors for **(A)** cervical cancer, **(B)** endometrial cancer, and **(C)** ovarian cancer. Box plots show the natural logarithm of (1 + expression value) of TCGA-CESC, TCGA-UCEC, and TCGA-OV drawn from RTCGA.rnaseq package. ****p < 0.0001.

To identify molecular pathways that distinguish CC, EC, and OC, we selected transcription factor (TF) genes that are exclusively activated/suppressed (amplified/deleted) for each cancer type in the Chinese cohort. Then we used the corresponding TCGA transcriptomic data to verify whether there are significant differences in expression of these TFs and their target genes among the three gynecologic cancers. Here we define a candidate TF as “exclusively altered” if its expression value in the corresponding cancer type exceed 1.5 (or -1.5) fold-change to the other two cancer types. Pathways deemed significantly and exclusively altered for each cancer type were summarized in **Table 1**. We found 3q amplifications as a signature of CC samples, which resulted in SOX2 (3q26.33), TP63 (3q28), SHOX2 (3q25.32), EAF2 (3q13.33) amplifications in CHI-CC; these genes were also proved significantly over-expressed in TCGA-CESC (**Figure 3A**) as compared with TCGA-UCEC and TCGA-OV. The Sox2-p63 complex is known to promote tumor cell survival through up-regulation of GLUT1 (SLC2A1) that drives glucose influx to empower antioxidant production (18); the Sox2-p63-klf5 complex has been shown to enhance tumor growth by activation of ALDH3A1 (19). Overexpression of these effector genes within the sox2-p63 pathways in TCGA-CESC as compared with TCGA-UCEC and TCGA-OV are shown in **Supplementary Figure S1**. Shox2 has been reported as an epithelial-to-mesenchymal transition (EMT) inducer by up-regulating transforming growth factor β receptor I (TβR-I) expression (20). Eaf2 has been shown to activate Wnt3a signaling to protect cells from oxidative stress-induced apoptosis (21). The exclusive activation of Shox2-TβR-I and Eaf2-wnt3a in TCGA-CESC are also shown in **Supplementary Figure S1**. Two TFs, PBXIP1 and CREB3L4 (both at 1q21.3), were found exclusively amplified and over-expressed in EC (**Figure 3B**). Over-expression of PBXIP1 (HPIP) has been shown to inhibit apoptosis by up-regulating BCL2, to promote tumor cell proliferation via activation of ER, and to mediate EMT by regulating mesenchymal genes such as N-cadherin and Vimentin (22). The CREB3L4 transcription factor up-regulate the co-chaperone DNAJC12 (23), which has been proposed as a mediator of gastric cancer progression by regulating proliferation and invasion (24). **Supplementary Figure S2** shows the exclusive activation of PBXIP1-regulated genes and the CREB3L4-DNAJC12 axis in TCGA-UCEC. For OC (**Figure 3C**), we found exclusive amplifications and over-expression in HSF1 (8q24.3), FOXH1 (8q24.3), and ZFPM2 (8q23.1). Hsf1 is known as a master regulator in tumorigenesis that mediates cell survival and EMT via up-regulation of effector genes such as HSPA8 (hsp70), RMB23, and MTA1 (as validated in TCGA-OV, shown in **Supplementary Figure S3**) (25, 26). Foxh1 is a binding partner of Smad2/3/4 proteins and the Foxh1-Smads complex has been an activator of the Nodal signaling pathway that is required for maintenance of pluripotency (**Supplementary Figure S3**) (27–29). The ZFPM2 encodes the Fog2 (Friend of Gata, 2) protein, which can interact with Gata2/4/5/6. GATA6 has been shown to up-regulate expression of genes encoding important enzymes (e.g., CYP17A1) for androgen biosynthesis (30). We found over-expression of ZFPM2, GATA6, CYP17A1, and AR (androgen receptor) by TCGA-OV, as compared with TCGA-CESC and TCGA-UCEC (**Supplementary Figure S3**). All other exclusively altered TFs without known target/functional information are summarized in **Supplementary Figure S4**.

### Characteristics Shared Among Gynecologic Cancers, Validated by TCGA Data

Besides the above-mentioned common recurrent CNV events and a dMMR group found in each cancer type, comparative analysis with TCGA data revealed other commonly altered biological processes/pathways. We performed GO enrichment analysis (over-representation, FDR cutoff 0.05) on frequently mutated genes (altered in >= 5 samples) for CHI-CC, CHI-EC, CHI-OC, and their TCGA counterparts. Commonly enriched biological processes were listed in **Supplementary Table S3**. The large number of overlapping biological processes indicated similarity at a high level between the two populations and among cancer types. For example, genes within the PI3K-Akt-mTOR pathway were found commonly altered in each cancer type with more than 70% prevalence (**Supplementary Figure S5**).

**Figure 4:**
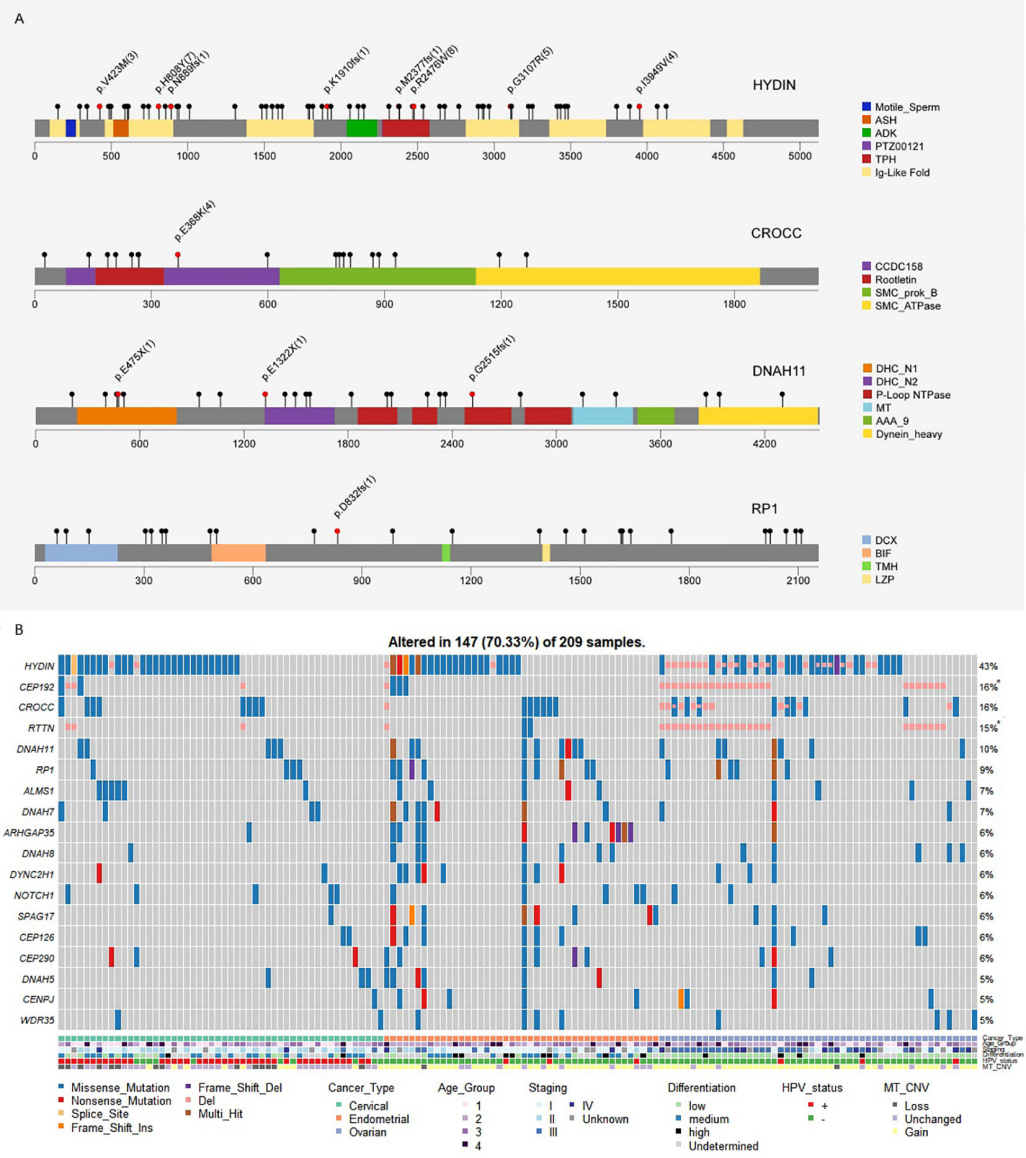
**(A)** Mutation spectra of the frequently altered cilia-related proteins. Scale bars represent length of the protein sequences, lollipops represent protein-altering mutations (excluding splice site/silent/UTR/intron/intergenic region mutations). Recurrent mutations (n≥3), nonsense and frameshift mutations are highlighted with text specifying amino acid changes and frequency (bracket). Functional domains are shown in different colors. **(B)** Cilium component genes are frequently altered in Chinese gynecologic cancers.

We also noticed an enrichment of frequently mutated genes associated with cilium organization (GO:0044782) and cilium or flagellum-dependent cell motility (GO:0001539), including top mutated genes HYDIN (43%), CROCC (16%), DNAH11 (10%), and RP1 (9%) (**Figure 1**; **Figure 4A**). More than 70% of samples in our Chinese cohort carried mutations in at least one cilia component gene (**Figure 4B**), and 56–66% of TCGA gynecologic cancer samples (only counting small-scale mutations) also carried mutations in these genes (**Supplementary Figure S6**). However, we noticed a difference at gene level (**Supplementary Figure S7**). The Chinese cohort (CHI-CC, CHI-EC, CHI-OC) showed a strong, centered preference in HYDIN, CROCC, and RP1 mutations, while their TCGA counterparts favored mutations in SYNE2, HTT, RTTN, and the dyneins (DNAH and DYNC) family genes, and having a more even distribution.

### Statistically Significant Prognostic Factors for Each Cancer Type

**Figure 5:**
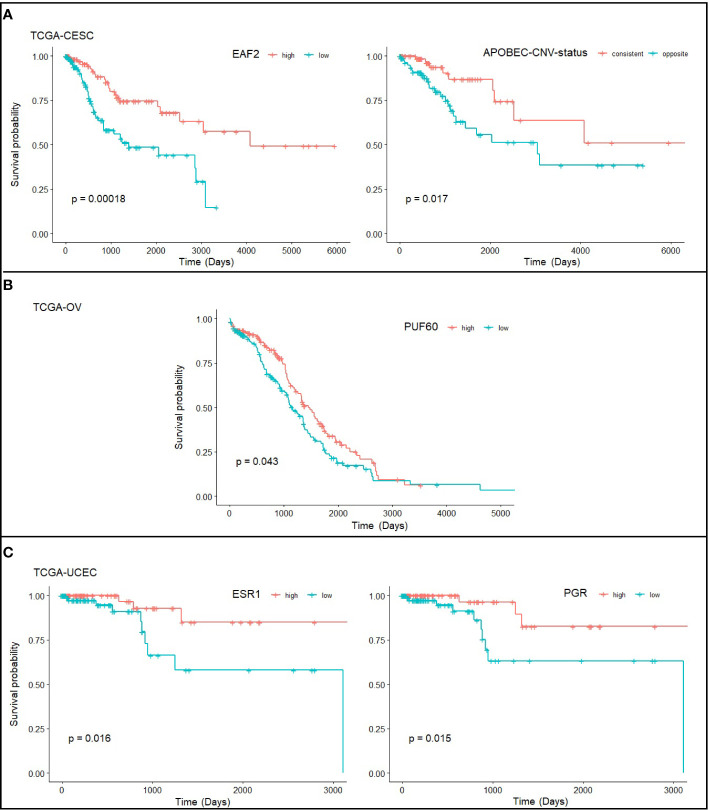
Statistically significant prognostic factors for **(A)** cervical cancer, **(B)** ovarian cancer, and **(C)** endometrial cancer. Survival plots show the correlation of gene expression level and survival probability of TCGA-CESC, TCGA-OV, and TCGA-UCEC drawn from RTCGA.rnaseq and RTCGA.clinical packages. Note that the TCGA-CESC study has defined the level of APOBEC mutagenesis (high/low) and CNV (high/low) level, and here we define “consistent” as consistently high or low in APOBEC and CNV, and “opposite” as inconsistent at APOBEC and CNV levels.

We further asked if expression level of exclusively altered genes for each cancer type is associated with survival. EAF2 was identified as a strong candidate for CC with p=0.00018, with low expression associated with poor prognosis. EAF2 has been proposed as a prognostic factor in prostate cancer (31). Interestingly, we found in the TCGA-CESC that APOBEC high/low (p=0.98) and CNV high/low (p=0.81) alone was statistically insignificant for predicting prognosis, but become significant (p=0.017) when combined together (**Figure 5A**), i.e., patients showing consistently high or low levels of APOBEC and CNV have better survival. PUF60 was one of the TFs exclusively up-regulated in OC (**Supplementary Figure S4**) and was found significantly associated with OC survival (p=0.043; **Figure 5B**). However, while a better outcome for PUF60 over-expressing OC patients was indicated in TCGA-OV transcriptomic data, others reported association of PUF60 over-expression with breast cancer progression through down-regulation of PTEN (32). Further verification about the roles of PUF60 in different cancers is awaited. ESR1 and PGR were found associated with patient survival in EC (**Figure 5C**), which is consistent with previous study (33).

## Discussion

We have performed a series of analysis to study the question of whether there are molecular characteristics shared/exclusive among gynecologic cancers, and more importantly to probe for the intrinsic causes of them. Using TCGA gynecologic cancer data as a validation, we confirmed that there are considerable similarities and differences among CC, EC, and OC, in frequently mutated genes, recurrent CNV events, affected molecular pathways, and biological processes. The molecular similarities shared among gynecologic cancers reflect the close proximity and functional connections among them, while the differences may reflect their distinct cell types of origin (e.g., squamous cells, serous cells, glandular cells).

The functions of the shared molecular features of gynecologic cancers reveal their associations with early tumor initiation. According to our results, all three gynecologic cancer types (both Chinese and TCGA) share a dMMR-signature, four recurrent CNV events, as well as the extensive alterations in PI3K-Akt-mTOR signaling and cilium component genes. It is well-established that accumulated genomic lesions caused by malfunction of DNA repair drive tumorigenesis (34), and that dMMR is viewed as a key inducer of tumor initiation. Mutant PIK3CA-induced constitutive PI3K activation has been shown to be essential for tumor initiation in mouse models of breast cancer (35) and able to dedifferentiate normal lineage-restricted cells by reactivation of multi-potency at early stage of tumor initiation (36). In line with early PI3K activation, ciliary defects have also been proposed with key roles in early stages of tumor development. Loss of primary cilia has been observed in breast pre-malignant lesions (37), and loss of motile cilia in Fallopian tube increases the exposure of epithelial cells to oxidative stress caused by follicular fluid (38). The recurrent CNV events shared by gynecologic cancers are predicted to cause amplifications of retinal proteins FAM138D and FAM138E, and deletions of genes associated with IFN-α/β signaling, which can be viewed as a strategy of immune evasion. Indeed, the immune system has been proven the ability of rapid sensing of oncogene-transformed cells (39); however, instead of effective killing, the tumor-associated immune cells may become protective upon interactions with the preneoplastic cells (40). These shared molecular changes suggest a common, non-random reprogramming of cells at the early stages of tumorigenesis. The reprogramming process involves changes in specific chromosome regions, resulting in up/down-regulation of genes with key roles in tumor biology, and through these alterations the preneoplastic cells become able to satisfy the minimal requirement for the establishment of a tumor. Future investigations are required to explore the potentials of the involved molecules as candidates for early biomarkers of gynecologic cancers.

Unlike the shared molecular changes that are associated with tumor initiation, the functions of exclusively altered pathways for each cancer type suggest roles in satisfying the needs for more advanced, later-stage cancer development, such as the maintenance of stemness, tumor growth, and migration. For CC, this may be at least partially powered by 3q amplifications that lead to the activation of the squamous lineage transcription factors Sox2 and p63, which are the master regulators of stem cell pluripotency (19); the activation of Shox2-TβR1 may serve as an inducer of EMT (20). There is a potential link between Sox2 amplification/over-expression and HPV-positivity in vulva cancer (41), which could also apply to the explanation of exclusive Sox2-p63 activation in CC. EC may handle these tasks by activation of PBXIP1/HPIP signalling and CREB3L4-DNAJC12 axis, while OC by Hsf1, Foxh1, and Fog2. Although different cancer types may activate/inactivate different TFs and pathways, eventually their consequences are similar (i.e., eventually achieving cell survival, proliferation, and EMT). This suggests that the downstream effector genes for various TFs could be overlapping or redundant, because they all eventually lead to cancer progression. These exclusively altered drivers indicate the existence of a cell-type specific developing trajectory, from which different types of pre-malignant cells gradually acquire cell-type specific molecular changes that eventually distinguish them (e.g., mutation load, CNV frequency, significantly altered genes, mutational signatures). The program may offer the ability of self-renewal and infinite proliferation, as well as the ability of tumor cell migration. Prognostic or diagnostic biomarker candidates for specific cancer type could be found within these exclusively altered molecules.

The molecular characteristics of Chinese gynecologic cancers can provide some implications for targeted therapies. More than 10% of the OV samples carried BRCA1/2 mutations, and some more with mutations in genes involved with homologous recombination repair, rending these patients potential sensitivity to PARP inhibitors, which is currently an available option for Chinese OV patients (42–44). Moreover, over 70% of the samples showed alterations in PI3K-Akt-mTOR signalling, which suggest great application potentials for PI3K/Akt/mTOR inhibitors.

Our results showed significant consistency with previous studies (2, 4). It is important to note that our study was based on tumor-only sequencing, i.e., no matched-normal samples were used. Such a condition represents a very common situation in the clinical setting where matched-normal samples were usually not available. One may question the reliability of the detected somatic variants for each individual sample because of the lack of normal control. We were fully aware of this concern and have added many extra filters (see Materials and Methods for details) to maximally avoid false positives; small-scale mutations of HYDIN were further validated with Sanger sequencing. The principle behind our study is the assumption that false discoveries occur randomly and their effects will be diluted if the sample size is large enough, while true mutations occur specifically on particular regions that will accumulate their effects as the sample size grows. The disadvantage of single-sample sequencing is thought to be negligible when focusing only on recurrent (>5% frequency) events. Indeed, the validation by TCGA data has proven the accuracy of our Chinese cohort data at the gene/pathway/process level. More efficient analytical tools are pending for the full exploitation of the large body of tumor-only samples.

In conclusion, we present here currently the largest molecular characterization of multiple types of Chinese gynecologic cancers. Using relevant TCGA data as a validation, we identified common molecular features among gynecologic cancers, which suggest a common reprogramming process of cells in early tumor initiation. We also identified exclusively altered TFs/pathways for CC, EC, and OC, which indicate a later-stage, cell-type specific tumor development process for each cancer type. From a molecular point of view, we have provided a summary of what is shared and what is not among gynecologic cancers and have given hypotheses about the causes behind these observations. Validations of our findings require further experimental research and large-scale cohort studies including multiple gynecologic cancer types.

## Data Availability Statement

The datasets presented in this study can be found in online repositories. The names of the repository/repositories and accession number(s) can be found below: https://bigd.big.ac.cn/gsa-human/browse/HRA000294.

## Ethics Statement

The studies involving human participants were reviewed and approved by the Ethics Committee of The Sixth Affiliated Hospital of Sun Yat-sen University. The patients/participants provided their written informed consent to participate in this study.

## Author Contributions

Conceptualization, SC and YS. Methodology, XY, TO, and ZL. Formal analysis, YG, JuL, JiL, HW, XiaomL. Resources, SC, YS, and XY. Data curation, YG, JuL, MiW, XiaoL, and MaW. Validation, MiW, XiaoL, and MaW. Writing—original draft preparation, YG, JuL, JiL. Writing—review and editing, JiL. Supervision, SC and YS. Project administration, SC and YS. All authors contributed to the article and approved the submitted version.

## Conflict of Interest

Authors JL, XY, HW, TO, XLia, ZL, and YS were employed by the company Top Gene Tech (Guangzhou) Co., Ltd.

The remaining authors declare that the research was conducted in the absence of any commercial or financial relationships that could be construed as a potential conflict of interest.

## References

[1] Bray F Ferlay J Soerjomataram I Siegel RL Torre LA Jemal A Global cancer statistics 2018: GLOBOCAN estimates of incidence and mortality worldwide for 36 cancers in 185 countries. CA Cancer J Clin (2018) 68(6):394–424. 10.3322/caac.21492 30207593

[2] Berger AC Korkut A Kanchi RS Hegde AM Lenoir W Liu W A Comprehensive Pan-Cancer Molecular Study of Gynecologic and Breast Cancers. Cancer Cell (2018) 33:690–705.e9. 10.1016/j.ccell.2018.03.014 29622464 PMC5959730

[3] Klotz DM Wimberger P Cells of origin of ovarian cancer: ovarian surface epithelium or fallopian tube? Arch Gynecol Obstet (2017) 296(6):1055–62. 10.1007/s00404-017-4529-z 28940023

[4] Wang M Fan W Ye M Tian C Zhao L Wang J Molecular profiles and tumor mutational burden analysis in Chinese patients with gynecologic cancers. Sci Rep (2018) 8:1–9. 10.1038/s41598-018-25583-6 29895933 PMC5997642

[5] Burk RD Chen Z Saller C Tarvin K Carvalho AL Scapulatempo-Neto C Integrated genomic and molecular characterization of cervical cancer. Nature (2017) 543:378–84. 10.1038/nature21386 PMC5354998 28112728

[6] Getz G Gabriel SB Cibulskis K Lander E Sivachenko A Sougnez C Integrated genomic characterization of endometrial carcinoma. Nature (2013) 497:67–73. 10.1038/nature12113 23636398 PMC3704730

[7] Bell D Berchuck A Birrer M Chien J Cramer DW Dao F Integrated genomic analyses of ovarian carcinoma. Nature (2011) 474:609–15. 10.1038/nature10166 PMC3163504 21720365

[8] Chandrani P Kulkarni V Iyer P Upadhyay P Chaubal R Das P NGS-based approach to determine the presence of HPV and their sites of integration in human cancer genome. Br J Cancer (2015) 112:1958–65. 10.1038/bjc.2015.121 PMC4580395 25973533

[9] Chen S Zhou Y Chen Y Gu J fastp: an ultra-fast all-in-one FASTQ preprocessor. Bioinformatics (2018) 34(17):i884–90. 10.1093/bioinformatics/bty560 PMC6129281 30423086

[10] Li H Durbin R Fast and accurate short read alignment with Burrows-Wheeler transform. Bioinformatics (2009) 25:1754–60. 10.1093/bioinformatics/btp324 PMC2705234 19451168

[11] McKenna A Hanna M Banks E Sivachenko A Cibulskis K Kernytsky A The Genome Analysis Toolkit: a MapReduce framework for analyzing next-generation DNA sequencing data. Genome Res (2010) 20:1297–303. 10.1101/gr.107524.110 PMC2928508 20644199

[12] Li H Handsaker B Wysoker A Fennell T Ruan J Homer N The Sequence Alignment/Map format and SAMtools. Bioinformatics (2009) 25:2078–9. 10.1093/bioinformatics/btp352 PMC2723002 19505943

[13] Smith KS Yadav VK Pei S Pollyea DA Jordan CT De S SomVarIUS: somatic variant identification from unpaired tissue samples. Bioinformatics (2016) 32:808–13. 10.1093/bioinformatics/btv685 26589277

[14] Lawrence MS Stojanov P Polak P Kryukov GV Cibulskis K Sivachenko A Mutational heterogeneity in cancer and the search for new cancer-associated genes. Nature (2013) 499:214–8. 10.1038/nature12213 PMC3919509 23770567

[15] Mermel CH Schumacher SE Hill B Meyerson ML Beroukhim R Getz G GISTIC2.0 facilitates sensitive and confident localization of the targets of focal somatic copy-number alteration in human cancers. Genome Biol (2011) 12:R41. 10.1186/gb-2011-12-4-r41 21527027 PMC3218867

[16] Mayakonda A Lin DC Assenov Y Plass C Koeffler HP Maftools: Efficient and comprehensive analysis of somatic variants in cancer. Genome Res (2018) 28:1747–56. 10.1101/gr.239244.118 PMC6211645 30341162

[17] Liao Y Wang J Jaehnig EJ Shi Z Zhang B WebGestalt 2019: gene set analysis toolkit with revamped UIs and APIs. Nucleic Acids Res (2019) 47:W199–205. 10.1093/nar/gkz401 PMC6602449 31114916

[18] Hsieh MH Choe JH Gadhvi J Kim YJ Arguez MA Palmer M p63 and SOX2 Dictate Glucose Reliance and Metabolic Vulnerabilities in Squamous Cell Carcinomas. Cell Rep (2019) 28:1860–78.e9. 10.1016/j.celrep.2019.07.027 31412252 PMC7048935

[19] Jiang Y-Y Jiang Y Li C-Q Zhang Y Dakle P Kaur H TP63, SOX2 and KLF5 Establish Core Regulatory Circuitry and Construct Cancer Specific Epigenome in Esophageal Squamous Cell Carcinoma. Gastroenterology (2020) 159(4):1311–27.e19. 10.1053/j.gastro.2020.06.050 32619460

[20] Hong S Noh H Teng Y Shao J Rehmani H Ding HF SHOX2 is a direct miR-375 target and a novel epithelial-to-mesenchymal transition inducer in breast cancer cells. Neoplasia (2014) 16:279–290.e5. 10.1016/j.neo.2014.03.010 24746361 PMC4094831

[21] Feng K Guo HK Eaf2 protects human lens epithelial cells against oxidative stress-induced apoptosis by Wnt signaling. Mol Med Rep (2018) 17:2795–802. 10.3892/mmr.2017.8246 PMC5783493 29257273

[22] Feng Y Xu X Zhang Y Ding J Wang Y Zhang X HPIP is upregulated in colorectal cancer and regulates colorectal cancer cell proliferation, apoptosis and invasion. Sci Rep (2015) 5:1–11. 10.1038/srep09429 PMC4371107 25800793

[23] Choi J Djebbar S Fournier A Labrie C The co-chaperone DNAJC12 binds to Hsc70 and is upregulated by endoplasmic reticulum stress. Cell Stress Chaperones (2014) 19:439–46. 10.1007/s12192-013-0471-6 PMC3982032 24122553

[24] Uno Y Kanda M Miwa T Umeda S Tanaka H Tanaka C Increased Expression of DNAJC12 is Associated with Aggressive Phenotype of Gastric Cancer. Ann Surg Oncol (2019) 26:836–44. 10.1245/s10434-018-07149-y 30617870

[25] Mendillo ML Santagata S Koeva M Bell GW Hu R Tamimi RM HSF1 drives a transcriptional program distinct from heat shock to support highly malignant human cancers. Cell (2012) 150:549–62. 10.1016/j.cell.2012.06.031 PMC3438889 22863008

[26] Calderwood SK HSF1, A Versatile Factor in Tumorogenesis. Curr Mol Med (2012) 12:1102–7. 10.2174/156652412803306675 PMC4075975 22804234

[27] Takahashi K Tanabe K Ohnuki M Narita M Sasaki A Yamamoto M Induction of pluripotency in human somatic cells via a transient state resembling primitive streak-like mesendoderm. Nat Commun (2014) 5:1–9. 10.1038/ncomms4678 24759836

[28] Chiu WT Le RC Blitz IL Fish MB Li Y Biesinger J Genome-wide view of TGFβ/Foxh1 regulation of the early mesendoderm program. Development (2014) 141:4537–47. 10.1242/dev.107227 PMC4302925 25359723

[29] Vallier L Mendjan S Brown S Ching Z Teo A Smithers LE Activin/Nodal signalling maintains pluripotency by controlling Nanog expression. Development (2009) 136:1339–49. 10.1242/dev.033951 PMC2687465 19279133

[30] Pihlajoki M Färkkilä A Soini T Heikinheimo M Wilson DB GATA factors in endocrine neoplasia. Mol Cell Endocrinol (2016) p:2–17. 10.1016/j.mce.2015.05.027 PMC4662929 26027919

[31] Zang Y Dong Y Yang D Xue B Li F Gu P Expression and prognostic significance of ELL-associated factor 2 in human prostate cancer. Int Urol Nephrol (2016) 48:695–700. 10.1007/s11255-015-1210-y 26895851 PMC4911596

[32] Sun D Lei W Hou X Li H Ni W PUF60 accelerates the progression of breast cancer through downregulation of PTEN expression. Cancer Manag Res (2019) 11:821–30. 10.2147/CMAR.S180242 PMC6340502 30697074

[33] Guan J Xie L Luo X Yang B Zhang H Zhu Q The prognostic significance of estrogen and progesterone receptors in grade I and II endometrioid endometrial adenocarcinoma: Hormone receptors in risk stratification. J Gynecol Oncol (2019) 30:1–14. 10.3802/jgo.2019.30.e13 PMC6304404 30479097

[34] Kiwerska K Szyfter K DNA repair in cancer initiation, progression, and therapy—a double-edged sword. J Appl Genet (2019) p:329–34. 10.1007/s13353-019-00516-9 PMC6803590 31468363

[35] Sheen MR Marotti JD Allegrezza MJ Rutkowski M Conejo-Garcia JR Fiering S Constitutively activated PI3K accelerates tumor initiation and modifies histopathology of breast cancer. Oncogenesis (2016) 5:e267–11. 10.1038/oncsis.2016.65 PMC5141269 27797363

[36] Van Keymeulen A Lee MY Ousset M Brohée S Rorive S Giraddi RR Reactivation of multipotency by oncogenic PIK3CA induces breast tumour heterogeneity. Nature (2015) 525:119–23. 10.1038/nature14665 26266985

[37] Menzl I Lebeau L Pandey R Hassounah NB Li FW Nagle R Loss of primary cilia occurs early in breast cancer development. Cilia (2014) 3:7. 10.1186/2046-2530-3-7 24987519 PMC4076761

[38] Coan M Vinciguerra GLR Cesaratto L Gardenal E Bianchet R Dassi E Exploring the role of fallopian ciliated cells in the pathogenesis of high-grade serous ovarian cancer. Int J Mol Sci (2018) 19(9):2512. 10.3390/ijms19092512 PMC6163198 30149579

[39] Feng Y Santoriello C Mione M Hurlstone A Martin P Live imaging of innate immune cell sensing of transformed cells in zebrafish larvae: Parallels between tumor initiation and wound inflammation. PloS Biol (2010) 8:e1000562. 10.1371/journal.pbio.1000562 21179501 PMC3001901

[40] Antonio N Bønnelykke-Behrndtz ML Ward LC Collin J Christensen IJ Steiniche T The wound inflammatory response exacerbates growth of pre-neoplastic cells and progression to cancer. EMBO J (2015) 34:2219–36. 10.15252/embj.201490147 PMC4585460 26136213

[41] Gut A Moch H Choschzick M SOX2 Gene Amplification and Overexpression is Linked to HPV-positive Vulvar Carcinomas. Int J Gynecol Pathol (2018) 37:68–73. 10.1097/PGP.0000000000000388 28700423

[42] Gallotta V Conte C D’Indinosante M Capoluongo E Minucci A De Rose AM Prognostic factors value of germline and somatic brca in patients undergoing surgery for recurrent ovarian cancer with liver metastases. Eur J Surg Oncol (2019) 45(11):2096–102. 10.1016/j.ejso.2019.06.023 31227342

[43] Gallotta V Bruno M Conte C Giudice MT Davià F Moro F Salvage lymphadenectomy in recurrent ovarian cancer patients: Analysis of clinical outcome and BRCA1/2 gene mutational status. Eur J Surg Oncol (2020) 46(7):1327–33. 10.1016/j.ejso.2020.01.035 32085925

[44] Póti Á Gyergyák H Németh E Rusz O Tóth S Kovácsházi C Correlation of homologous recombination deficiency induced mutational signatures with sensitivity to PARP inhibitors and cytotoxic agents. Genome Biol (2019) 20(1):240. 10.1186/s13059-019-1867-0 31727117 PMC6857305

