# Supplementary material for: Molecular Profiling Reveals Common and Specific Development Processes in Different Types of Gynecologic Cancers
Source: Front Oncol. 2020 Oct 29;10:584793. doi: 10.3389/fonc.2020.584793 (PMC7658613; doi:10.3389/fonc.2020.584793)
Supplement: Supplementary file 1 [file Table_1.docx]

Supplementary Material


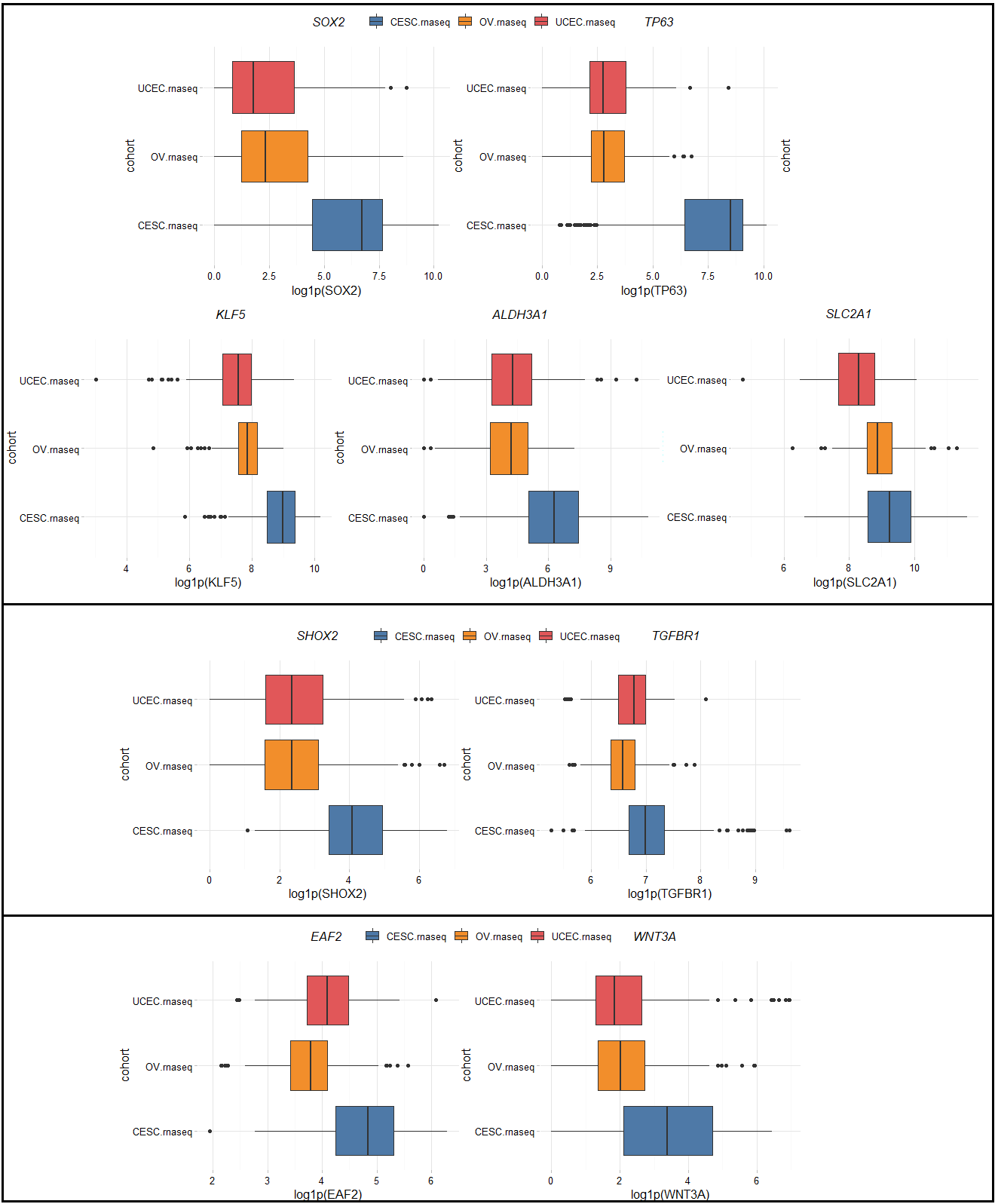


Figure S1: Exclusively altered transcription factors and their effector genes in cervical cancers.


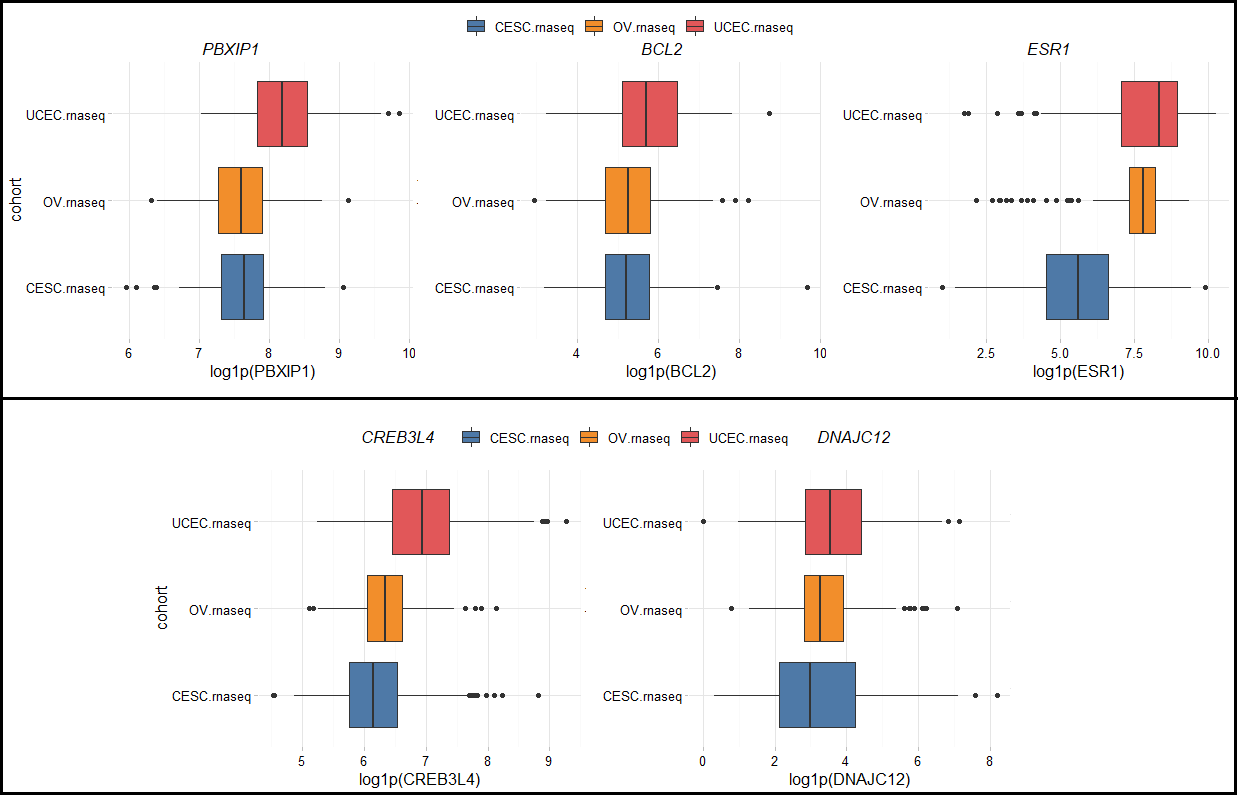


Figure S2: Exclusively altered transcription factors and their effector genes in endometrial cancers.


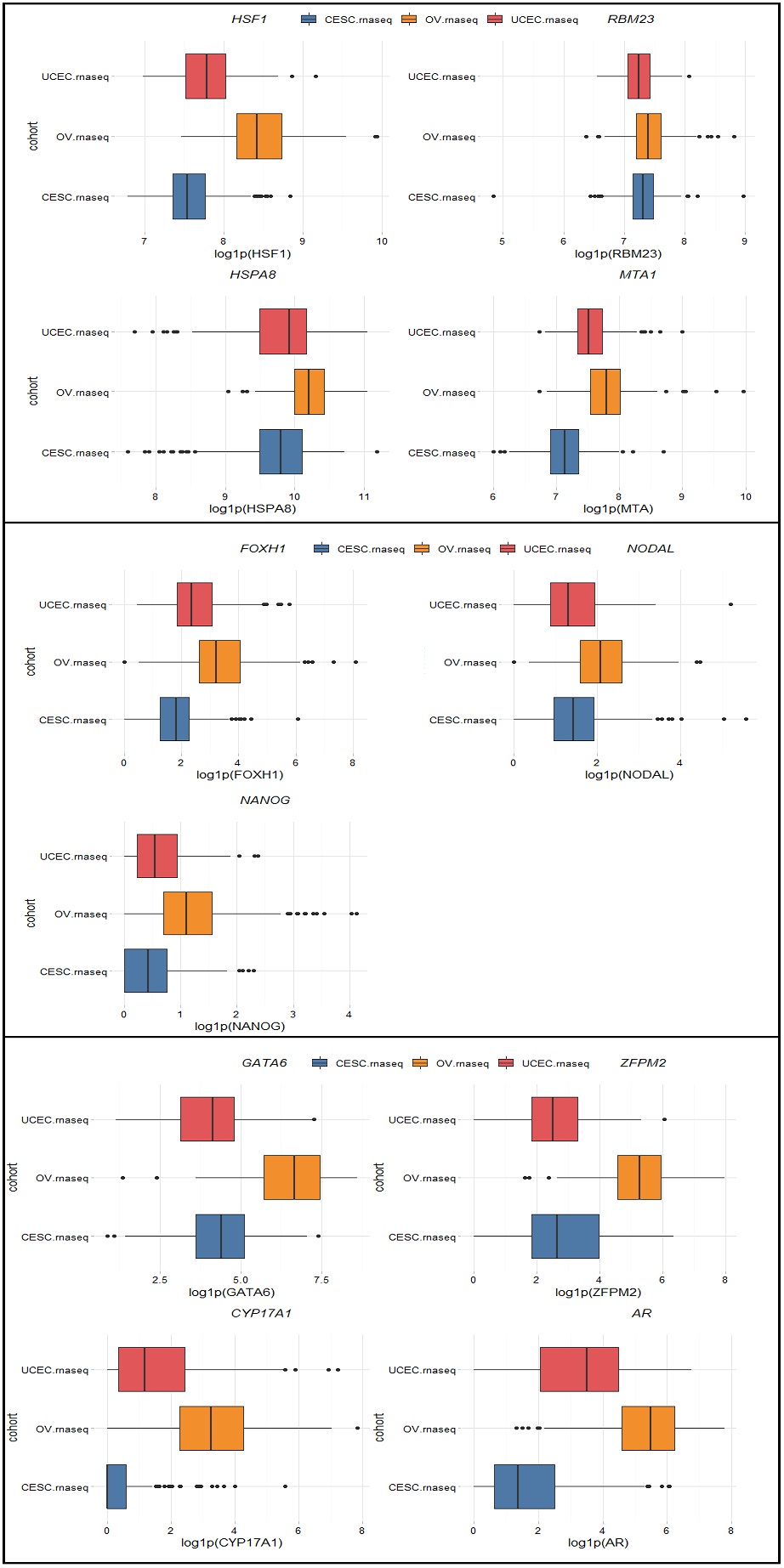


Figure S3: Exclusively altered transcription factors and their effector genes in ovarian cancers.


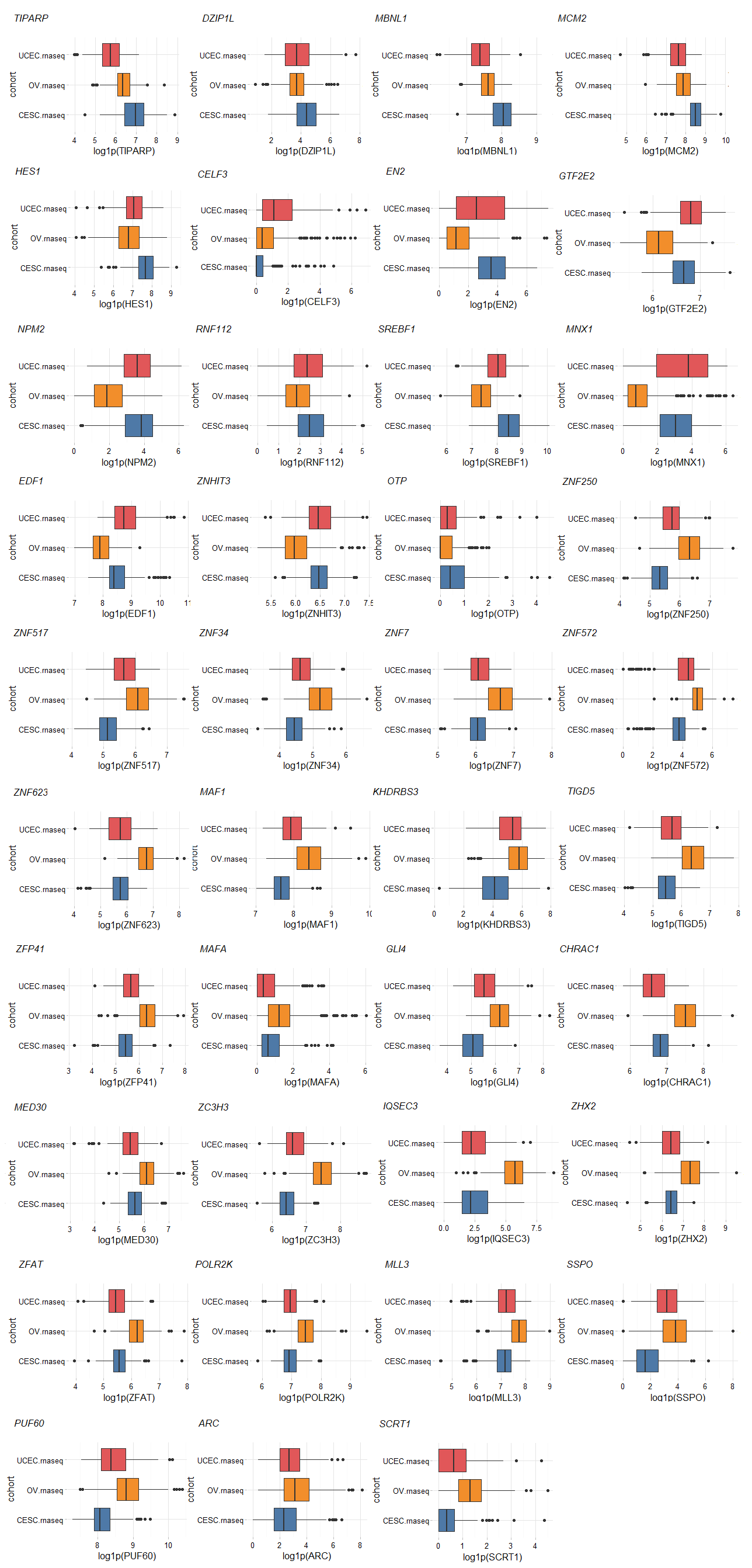


Figure S4: Exclusively altered transcription factors without known target/functional information.


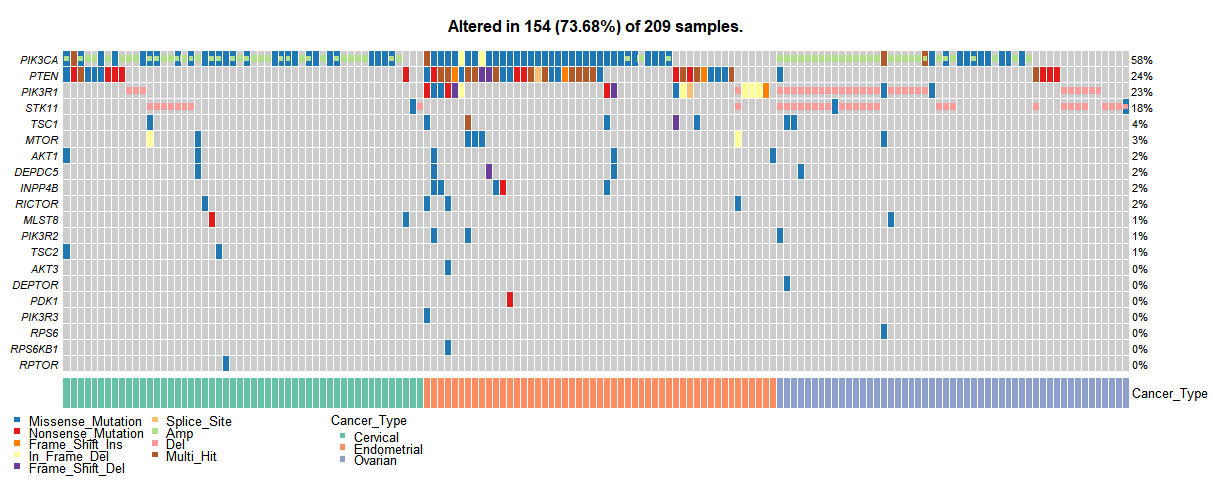


Figrue S5: Mutations in PI3K-Akt-mTOR pathway genes.


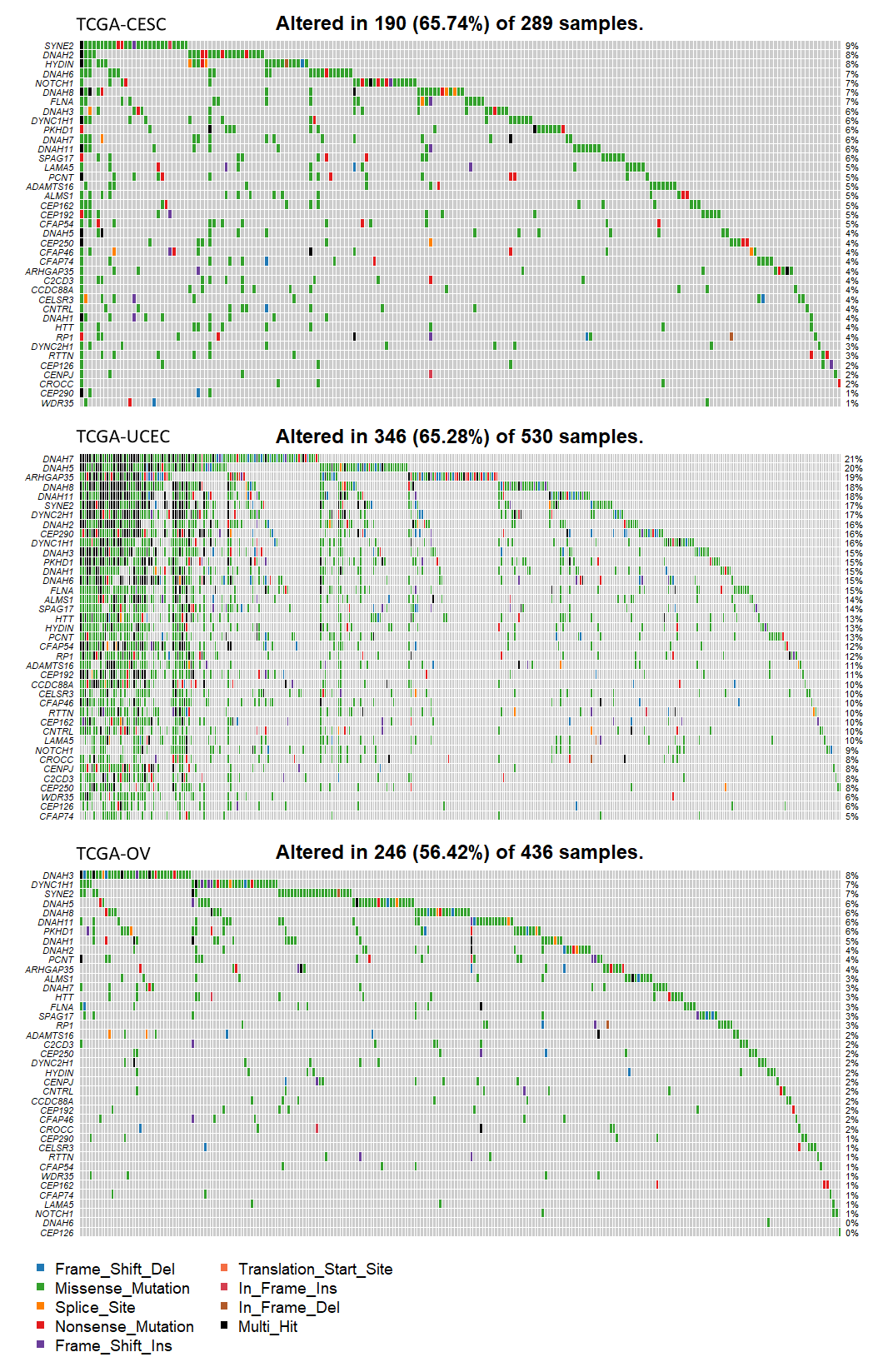


Figrue S6: Frequently mutated cilia organization genes in TCGA gynecologic cohorts.


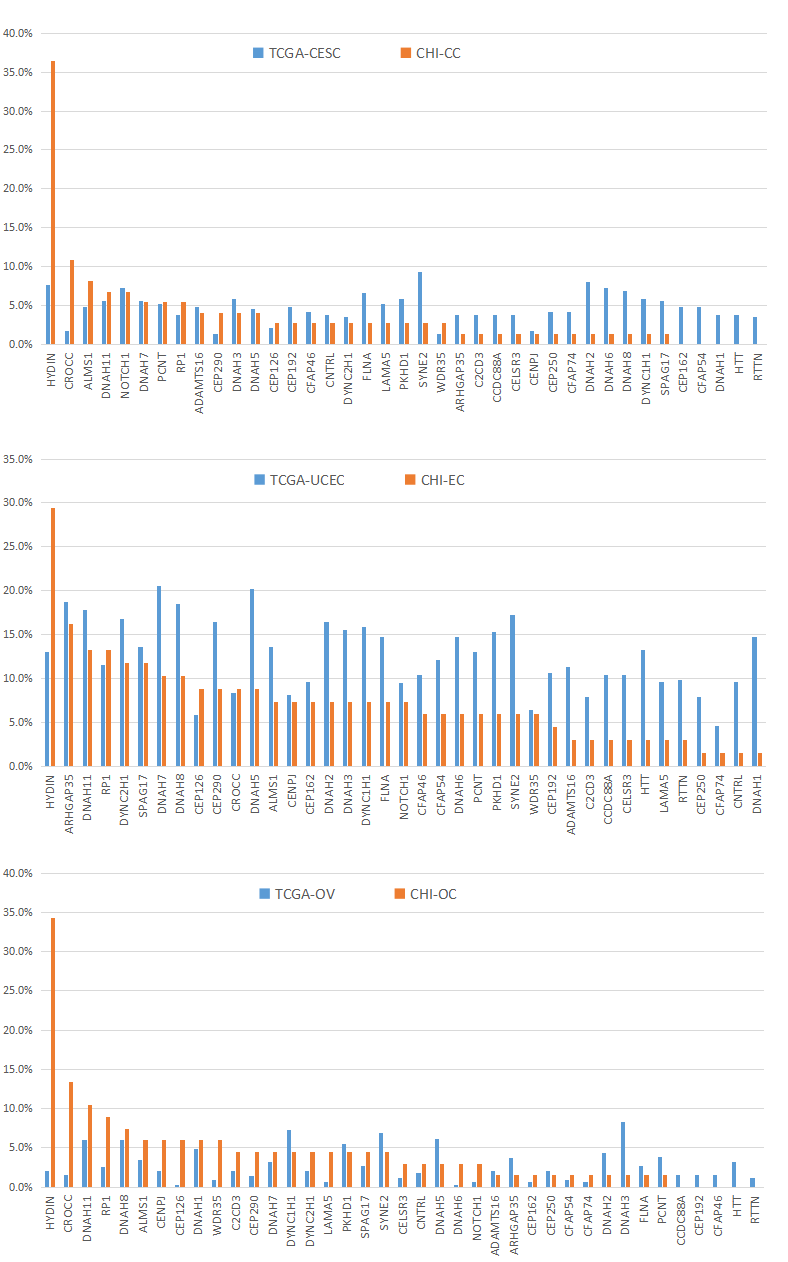


Figrue S7: Barplot comparison of frequently mutated cilia organization genes between Chinese (CHI) and TCGA gynecologic cancer samples.

Table S1:Demographics of the 209 Chinese gynecologic cancer patients.

Table S2: Significantly mutated genes as calculated by MutSigCV. Genes that are also significantly mutated in relevant TCGA cohorts are highlighted in red.


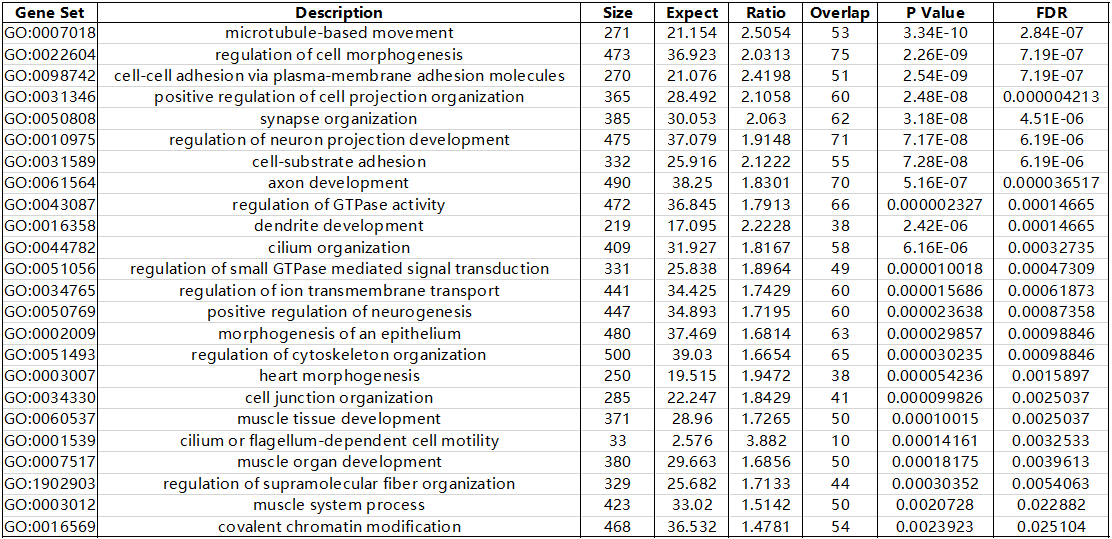


Table S3: Shared enriched biological processes frequently altered CC, EC and OC of the studied Chinese and TCGA gynecologic cancer cohorts.
